# Prognosis Prediction for Colorectal Cancer Patients: A Risk Score Based on The Metabolic-Related Genes

**DOI:** 10.7150/ijms.49576

**Published:** 2021-01-01

**Authors:** Yongqu Lu, Xin Zhou, Zhenzhen Liu, Wendong Wang, Siyi Lu, Wei Fu

**Affiliations:** Department of General Surgery, Peking University Third Hospital, Beijing, 100191, China.

**Keywords:** bioinformatics, colorectal cancer, metabolic-related gene, prediction model, risk score

## Abstract

Risk assessment has high prognostic value in patients with colorectal cancer (CRC), and the use of proper models is an effective approach frequently used to evaluate cancer progression for further treatment plans. Alterations in metabolism are confirmed to be a significant feature of tumor cells and have been an intense focus in disease research. Here, we mined the genes that were differentially expressed in CRC tissues compared to paired normal samples from a public database and then constructed a novel assessment system for the prognosis of patients based on the value of a risk score considering the expression status of metabolism-related genes after screening. The score successfully stratified patients by risk and was externally verified in our study. Moreover, we built a nomogram combining the score and clinical parameters to predict patient survival using a visual method. The results suggested that the risk score was well fit and could provide assistance for the individual treatment of CRC patients in the clinic.

## Introduction

Colorectal cancer (CRC), a malignant tumor of the digestive tract, commonly occurs worldwide. According to the estimated global cancer statistics, CRC ranked third in terms of incidence and second in terms of mortality in 2018 1. Treatment and prognosis vary widely for patients with different cancer stages and biological features. Based on tumor-node-metastasis (TNM) staging, approximately one in five CRC patients diagnosed with stage I has a five-year survival rate over 90%, while the survival declines to 12% for stage IV CRC, which is of clinical significance for patients and decision-makers to consider 2. Suitable methods and tools to predict survival prognosis are being explored and developed. The measurement of carcinoembryonic antigen levels in blood is recommended as a routine test at the time of diagnosis and postoperative period, and elevated concentrations may indicate a poor prognosis 3. However, due to the complexity of the disease, the results of a single test tend to have low accuracy. Therefore, a more comprehensive method for risk stratification is of clinical significance.

Alterations in nutrient and energy metabolism are closely involved in fueling tumor growth and division 4. Glucose, lipids and proteins in the processes of transformation or transportation affect the maturation and differentiation of invasive tumor cells. The rules of metabolism in tumor tissues may significantly differ from those in normal tissues, such as the Warburg effect, because of adaptative reprogramming 5,6. In addition to metabolites, several related signaling pathways have been indicated to participate in the intervention of proliferation and are associated with activated oncogenes in tumors 7. By measuring the dynamic changes in metabolites from samples such as blood and tumor tissues, metabolomics is able to determine the pathophysiological state of CRC patients to a certain extent, and currently, some biomarkers have been identified to have potential prognostic value 8,9. Reprogramming metabolism as a promising target for initial prevention and selected treatment along with the detection of metabolic alterations for risk assessment in CRC patients have been prospectively proposed 10.

In this study, we aimed to construct a prediction model for CRC patients concentrated on the expression of metabolism-related genes. We obtained the expression of genes from public databases and screened out potential genes with significant clinical value. Using these genes, we developed a prognostic scoring system to predict the risk of CRC patients and verified the accuracy with external data. Through statistical approaches, a novel model combining a metabolic risk score and clinical parameters was established. This model of CRC can guide genetic risk assessment and provide prognostic information for patients as well as lead to new strategies for therapeutic intervention.

## Materials and Methods

### Data extraction

For CRC patients, expression profiles including RNA-sequencing data from The Cancer Genome Atlas (TCGA) and two microarray datasets (GSE17538 and GSE39582 based on platform GPL570) from the Gene Expression Omnibus (GEO) database as well as corresponding clinical data were available 11,12. Only patients with a definite diagnosis of CRC and no less than 30 days of overall survival (OS) were included in the construction of the model. Furthermore, patients without extractable clinical parameters on age, sex and TNM stage were removed from the correlation testing of clinical factors. Calibrations to the same level and log2 transformations were performed for all expression data using an R package. The lists of metabolism-related genes were retrieved from Kyoto Encyclopedia of Genes and Genomes (KEGG) gene sets in the Molecular Signatures Database according to metabolism-related pathways 13. The data were identified and downloaded on January 15, 2020. In the TCGA dataset, 439 CRC patients were included for survival analysis, among which 426 patients had full clinical parameters. 229 cases of GSE17538 and 522 of GSE39582 met the requirement for validation.

### Construction of the risk score

The CRC samples from TCGA were used as the training cohort, and the differentially expressed metabolic genes related to prognosis were selected with limma package at a threshold of log_2_ fold change (FC) of 1.5 and false discovery rate (FDR) of 0.05. The corresponding coefficients for different metabolic genes in the model were confirmed after statistical estimation with glmnet package. A formula for the risk score of the model was established to predict patients' prognosis: risk score = Σ coefficient of gene *i* * expression value of gene *i.*

### Analysis of the prognostic genes

CRC samples with mutation and copy-number alteration (CNA) data in TCGA PanCancer Atlas study were analyzed in the online database cBioPortal 14. The validation utilized mRNA profile for comparison between the normal samples and tumor samples in another public database, Oncomine, and the threshold was set as follows: P-value, 1E-4; FC, 2; gene level, top 10% 15.

### Validation of the risk score

CRC patients with sufficient expression data and clinical information from the two GEO sets were used as the validation cohorts. The sva package was used for batch normalization. According to the median value of the risk score in the training cohort, the patients in the two cohorts were divided into two groups: high-risk and low-risk groups. Gene set enrichment analysis (GSEA, version 4.0.3) was performed to ascertain the correlation between the risk assessment and the metabolic genes, and the predictive value of the scores was tested based on survival curves, risk curves and receiver operating characteristic (ROC) curves with survival and survivalROC package. The process was carried out in accordance with the Transparent Reporting of a Multivariable Prediction Model for Individual Prognosis or Diagnosis (TRIPOD) statement (Table S1) 16.

### Construction of the nomogram

Independent risk factors were screened out using all patients from the training cohort and validation cohorts. Based on the identified variables, a nomogram was constructed for predicting one-, three- and five-year OS and visualizing the prognostic value with rms package.

### Statistical analysis

All statistical analyses were performed in R (version 3.6.0). The Wilcoxon test was used to identify genes differentially expressed in tumor samples and normal samples. Univariate Cox proportional hazards regression was used to estimate hazard ratios (HRs). Coefficients of the prediction model were generated by least absolute shrinkage and selection operator (LASSO) regression. The survival curve was generated by the Kaplan-Meier method, and the difference in OS was evaluated by the log-rank test. Univariate Cox analysis and multivariable Cox analysis were carried out to determine independent risk factors for the prognosis of CRC patients. Confidence intervals (CIs) were set at 95%, and a *P*-value <0.05 was considered significant in all statistical analyses.

## Results

### Screening of metabolism-related genes

**Figure 1:**
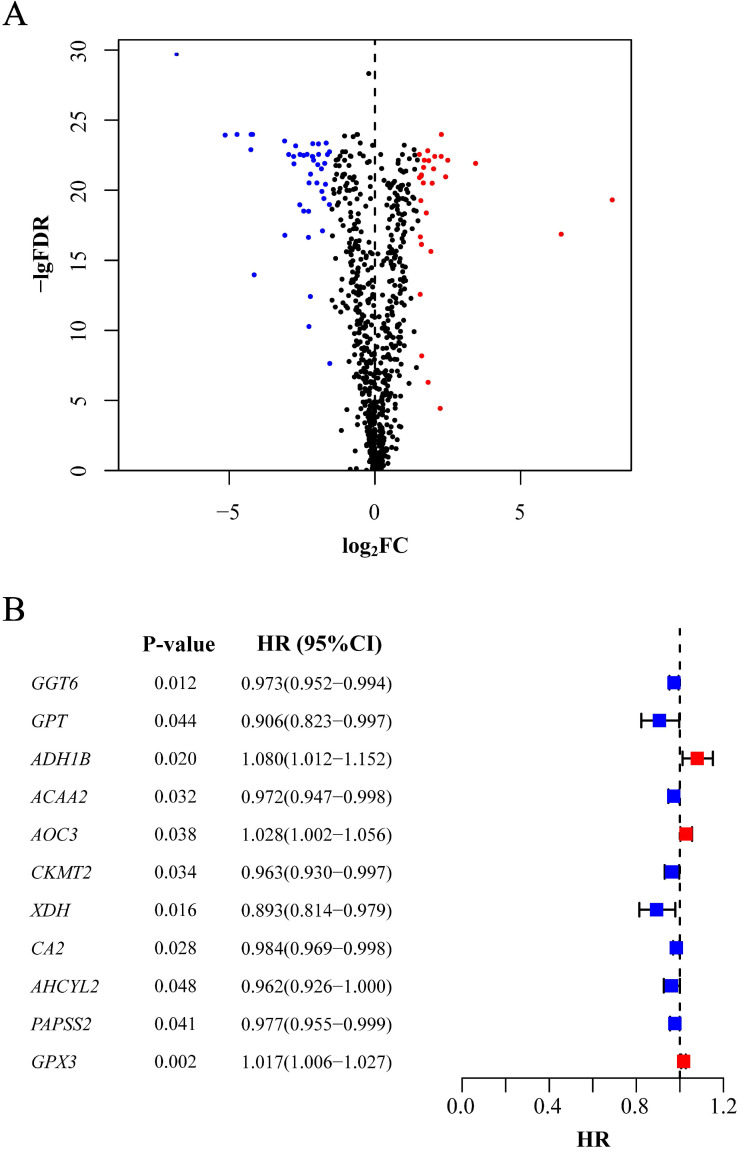
** Selection of candidate genes.** (A) Differentially expressed genes between tumor tissues and normal tissues. The red point stands for the upregulated gene and the blue for downregulated gene. Gene without significance is marked in black. (B) Genes significantly associated with prognosis after secondary screening. The red point stands for the HR of corresponding gene higher than 1 and the blue point for HR less than1. FDR: false discovery rate; FC: fold change; HR: hazard ratio; CI: confidence interval.

In total, 488 tumor samples and 42 normal samples from the training cohort were selected for gene expression analysis. We obtained 944 metabolism-related genes from KEGG gene sets, and differentially expressed genes were filtered based on the expression profiles, of which 43 were upregulated and 29 were downregulated in tumor samples (*P*<0.05, Figure 1A). For further selection, 11 genes were shown to have prognostic significance according to the calculated HR values of CRC patients. All the genes were detected in both the training and validation cohorts. Among them, 3 genes (*ADH1B*, *AOC3* and *GPX3*) might indicate worse prognosis than the other 8 genes (*P*<0.05, Figure 1B).

### Constructing the prediction model

Above, we had already obtained candidate prognosis-related metabolic genes. In consideration of the selected genes, we performed LASSO regression to build the model and identify the coefficients. Finally, 7 genes were included in the model, and each coefficient numerically represented the weight of the expression. The individual risk score was calculated based on a combination of the expression status of the prognostic genes and their corresponding coefficients (*P*<0.05, Table 1).

### Analysis of the prognostic genes in risk score

**Figure 2:**
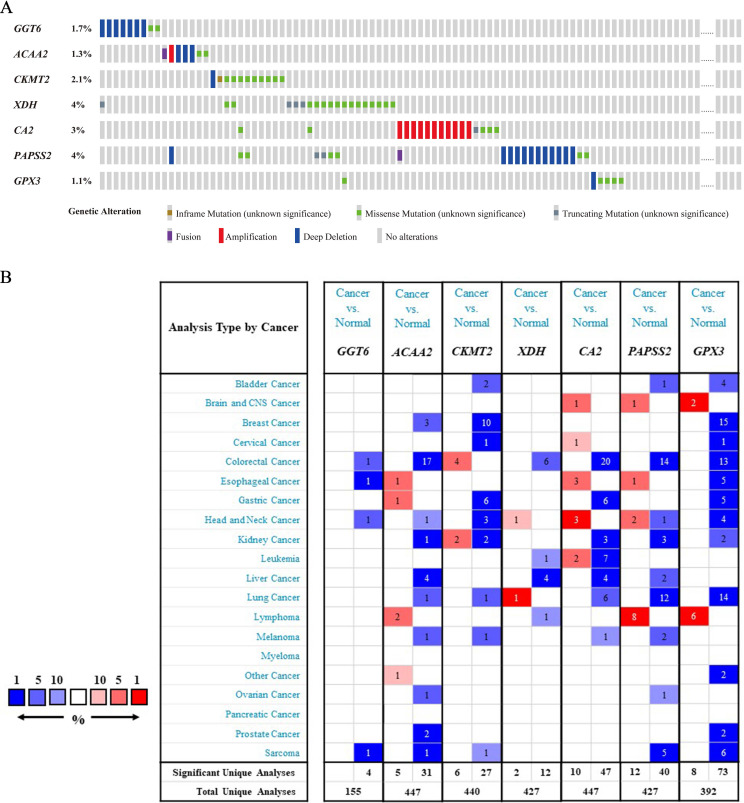
** Analysis of genes in risk score.** (A) Mutation and CNA data of CRC patients in cBioPortal database. The alteration rate for each gene is showed and different genetic alterations are marked with various colors. (B) Differential gene expressions in multiple cancer types from Oncomine database. The number in the cell represents the number of analyses meeting the threshold. Red represents a higher expression level of gene in tumor tissues comparing with normal tissues, and blue for the opposition.

In the 526 samples of cBioPortal dataset, *XDH* and *PAPSS2* had the most frequent mutation of 4% and the mutation of *GPX3* was the lowest in 1.1% samples (Figure 2A). Additionally, we found that the alterations of genes in the training cohort and the validation cohorts were coincident with the Oncomine dataset. The expressions of six genes were at low levels in CRC except for *CKMT2* (Figure 2B).

### Verification of the score

**Figure 3:**
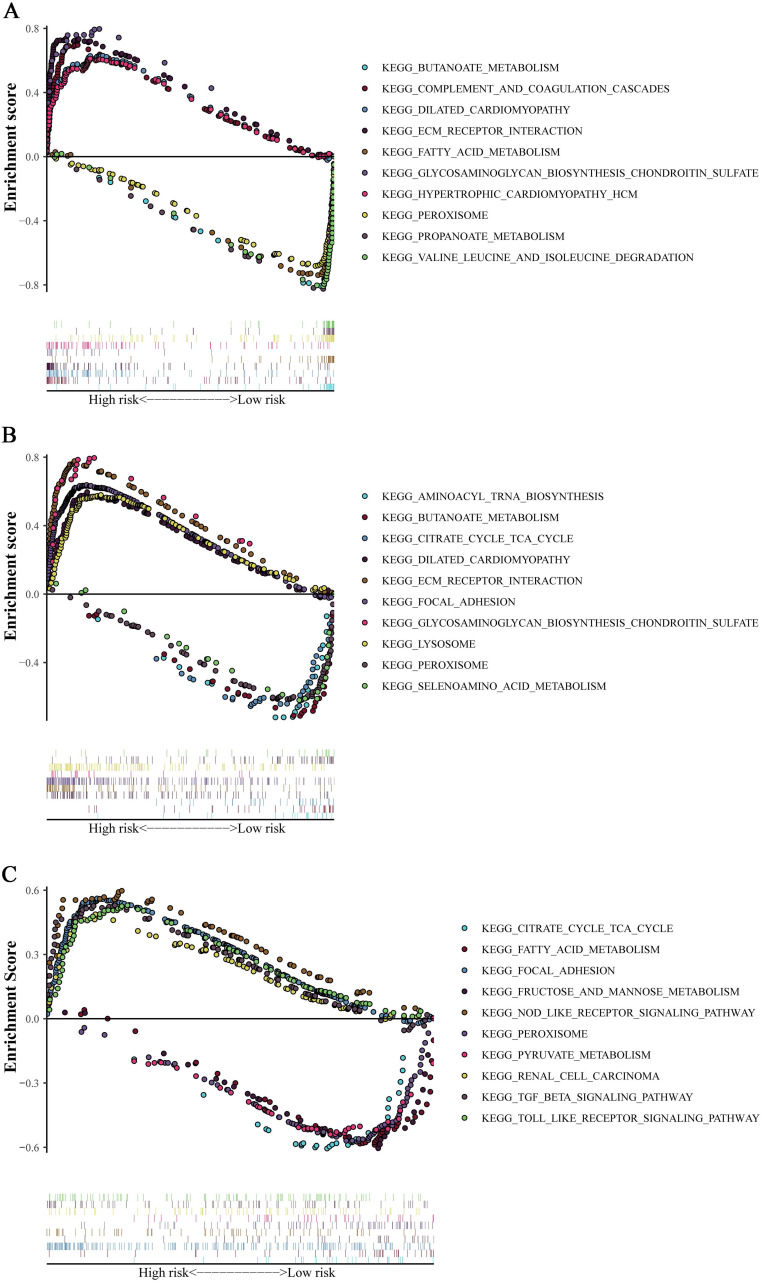
** GSEA analysis.** Pathway enrichment in training cohort (A) and validation cohorts (B for GSE17538 and C for GSE87211). The point indicates enrichment score of different cases in concentrative function.

To further confirm the reliability of the risk score, we simultaneously stratified the training cohort and the validation cohorts into high- and low-risk groups based on the median risk score in the training cohort. GSEA was performed to predict the pathways involved in the high-risk and low-risk groups, and the results showed that the risk score was significantly enriched in several metabolism-related biological processes (Figure 3).

**Figure 4:**
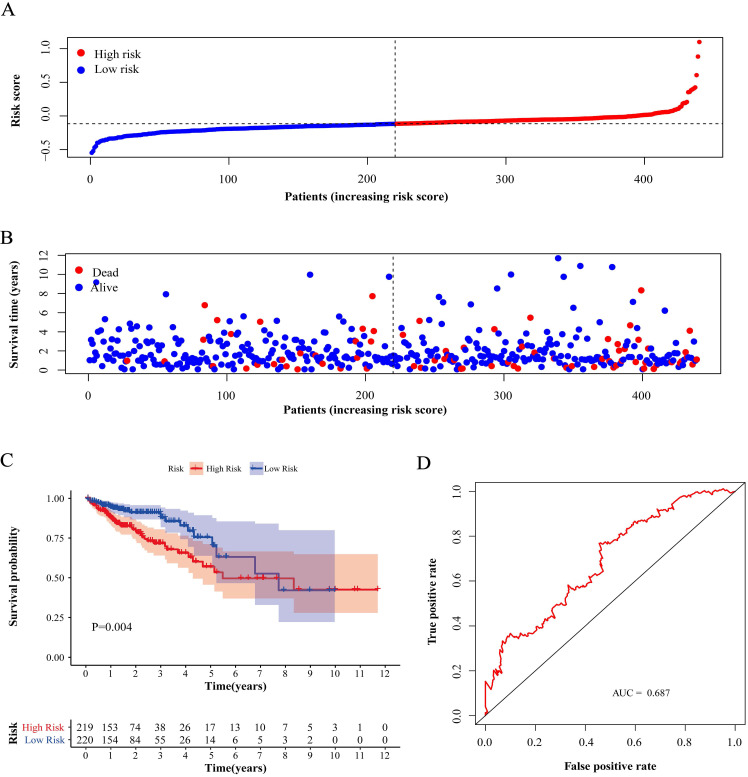
** Test of risk score in training cohort.** (A) Distribution of risk scores in high-risk group and low-risk group. Red point indicates case in high-risk group and blue indicates low-risk case. (B) Distribution of survival status of patients in high-risk group and low-risk group. Blue point represents alive and red point for death. (C) Survival curve of OS. Red line depicts the survival of high-risk patients and blue line for low-risk patients. (D) ROC Curve for risk score. AUC: area under curve.

**Figure 5:**
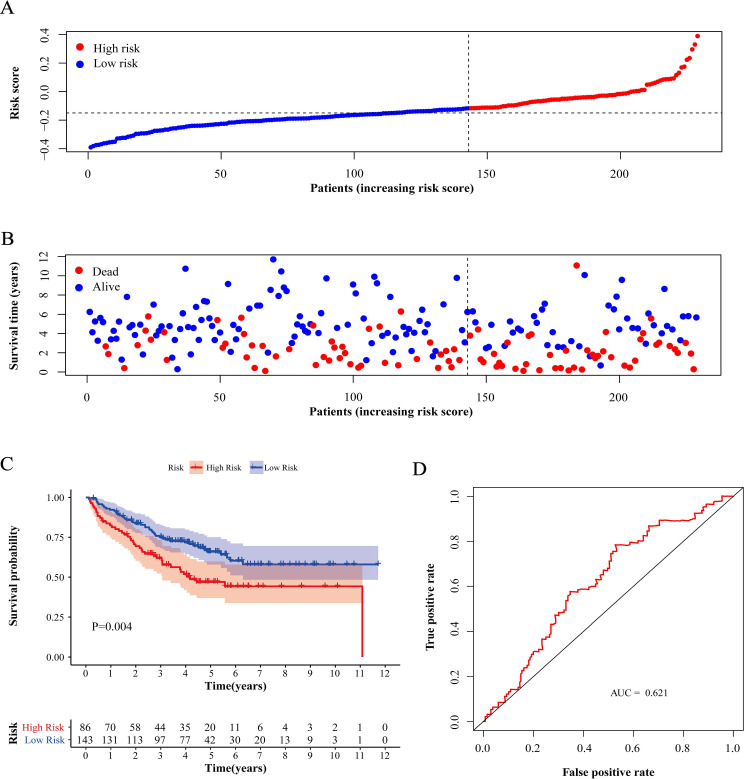
** Test of risk score in validation cohort (GSE17538).** (A) Distribution of risk scores in high-risk group and low-risk group. Red point indicates case in high-risk group and blue indicates low-risk case. (B) Distribution of survival status of patients in high-risk group and low-risk group. Blue point represents alive and red point for death. (C) Survival curve of OS. Red line depicts the survival of high-risk patients and blue line for low-risk patients. (D) ROC Curve for risk score. AUC: area under curve.

**Figure 6:**
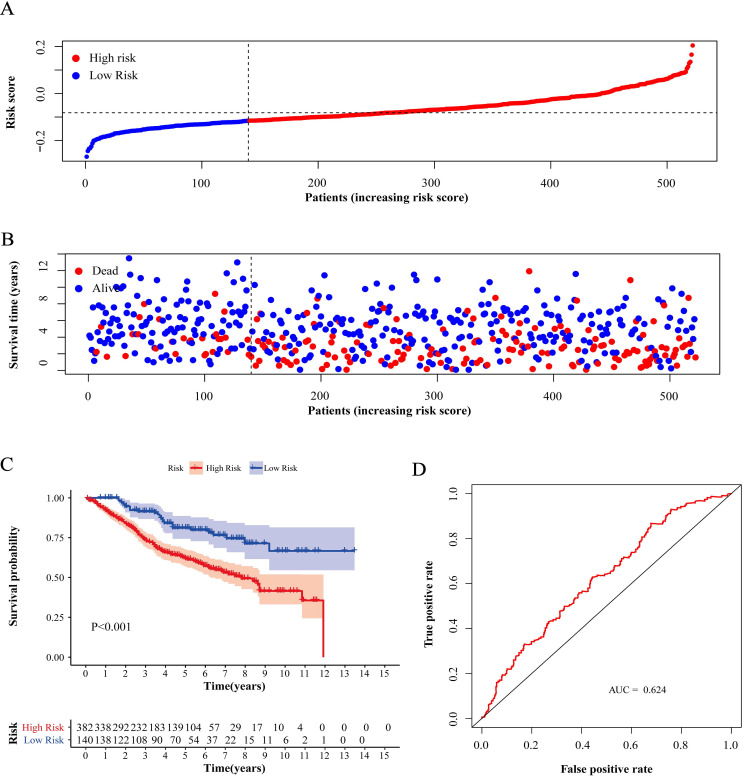
** Test of risk score in validation cohort GSE87211.** (A) Distribution of risk scores in high-risk group and low-risk group. Red point indicates case in high-risk group and blue indicates low-risk case. (B) Distribution of survival status of patients in high-risk group and low-risk group. Blue point represents alive and red point for death. (C) Survival curve of OS. Red line depicts the survival of high-risk patients and blue line for low-risk patients. (D) ROC Curve for risk score. AUC: area under curve.

Compared with low-risk patients, the worse prognosis of patients in the high-risk group was confirmed in both the training cohort and the validation cohorts based on the distribution of risk scores in low-risk group and high-risk group (Figure 4A, 5A and 6A) and the distribution of survival status of patients in low-risk group and high-risk group (Figure 4B, 5B and 6B). High-risk patients had a worse five-year OS than low-risk patients not only in the training cohort but also in the validation cohorts (P<0.05, Figure 4C, 5C and 6C). Additionally, the area under the curve (AUC) of the ROC curves of five-year survival for the training and validation cohorts were 0.687, 0.621 and 0.624 respectively, which indicated that the model had good effectiveness (Figure 4D, 5D and 6D).

### Evaluating the prognostic value of the risk score and constructing the nomogram for predicting survival

**Figure 7:**
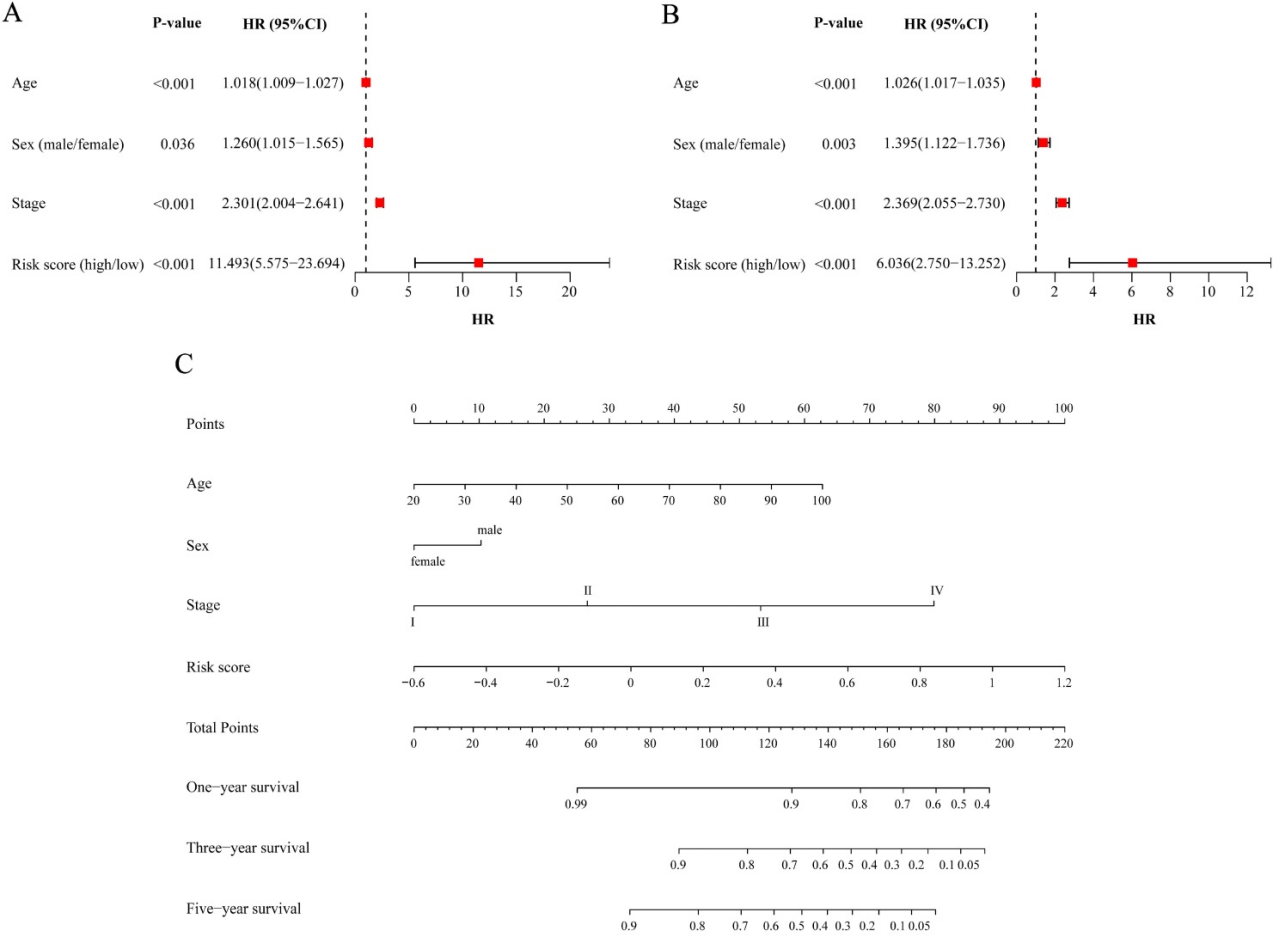
** Assessing risk factors of prognosis and constructing nomogram.** (A) Univariate Cox analysis of factors. (B) Multivariate Cox analysis of factors. (C) Estimation of survival through nomogram. One-year survival, three-year survival and five-year survival of corresponding point are showed in the lines. HR: hazard ratio; CI: confidence interval.

After we pooled all the data in the three cohorts together, the metabolic risk score and clinical parameters (age, sex and TNM stage) were included in the univariate analysis of patient survival to evaluate the predictive value of the model for prognosis. The results indicated that the age, sex, TNM stage and risk score of CRC patients were all correlated with prognosis (*P*<0.05, Figure 7A). The results of the multivariable analysis showed that age, sex, TNM stage and risk score could be independent predictive factors for patients (*P*<0.05, Figure 7B).

The independent predictive factors obtained from the multivariable analysis, including age, sex, TNM stage and risk score, were integrated to construct the nomogram for OS. A point scale was used to assign a value ranging from 0 to 100 for each variable, and the total points could be calculated to estimate the probability of survival at one, three and five years for CRC patients (Figure 7C).

## Discussion

In existing evaluation systems, predictive models for tumor prognosis mainly depend on clinical parameters considering the ease of obtaining patient data and subsequent assessment 17,18. With the development of gene detection technology, the sequencing of a panel of key genes or even whole genome sequencing can be implemented using patients' peripheral blood or biopsy tissues in hospitals or specialized laboratories. This allows genomics research to be more closely linked to clinical applications for diagnosis and treatment and makes the data construction of gene-related prediction practical. The prognosis of patients can be predicted more accurately at the genetic level, particularly with indicators that are specifically related to disease, and doctors can adopt an individualized approach for each patient. The study of the genome shows complex networks in genetic regulation, and a gene-related disease model concerning certain functions is quite reasonable.

Tumors have more complex metabolic changes termed metabolic reprogramming caused by mutations in oncogenes, which has been highlighted as a core hallmark of cancer 19. Metabolism, including catabolic processes and anabolic processes involving molecules such as lipid, glucose and protein, is involved in oncogenic pathways and affects the growth and proliferation of cancer cells. Production and utilization constitute a giant set of biochemical transformations in the urgent state of discovery 20. Considering the initiation and progression of CRC, metabolic markers can predict the response to individual therapy of patients and whether polyps will evolve into malignancy 21. Characteristic alterations in the metabolic process can occur much earlier than general symptoms in the clinic. The sequencing results of patients with familial adenomatous polyposis confirmed that metabolic reprogramming occurred at the stage of adenoma, early in the process of carcinogenesis 22. The application of metabolism-related genes has advantages in sensitivity and flexibility compared with whole genes or mRNA analysis 23. The promising prognostic value of the metabolism fingerprint for CRC patient survival has also been confirmed in various studies, which provides a substantial basis for our modeling work 24-26.

Here, we used public databases to identify metabolic genes related to CRC patient survival. A prediction model was constructed with the expression of 7 metabolism-related genes, and we assessed the applicability of this model with the external data. We validated the risk stratification with multiple methods and built a nomogram that utilizes clinical parameters for the visualization of survival prediction and is close to practical application. Almost all predictors incorporated into the model are associated with CRC according to our following findings from other independent studies. *CKMT2*, *CA2* and *GPX3* have been shown the association with the risk and survival of CRC patients, and *CA2* has a significant hazard ratio in elderly individuals 27-29. As an electron acceptor catalyzing the oxidation of purine, *XDH* was considered a key step in molecular mechanisms associated with purine metabolism in CRC through computational methods 30. Originating from the study of differential expression in metastatic and nonmetastatic CRC cell lines, *PAPSS2* was identified as a new molecular clone of 3'-phosphoadenosine 5'-phosphosulfate (PAPS) synthetase 31.

The predictive model in our study might have several limitations that potentially affect the results and need improvement. First, our data were extracted from public databases, and more data from a prospective study should be used to confirm the performance of the model and expand the scale of clinical cases. Second, we used the median risk score as the cutoff value for convenience, while the population-specific nature of the stratification must be considered for clinical application in practice, and the matched cutoff for specific patients should be fixed. The model needs adjustment for future applications. Third, the statistical method cannot discern the underlying mechanisms of interaction in selected genes and requires further exploration by molecular experiments.

In conclusion, risk score was constructed based on the expression status of prognosis-related metabolic genes and was validated in different situations. The nomogram including the risk score and clinical parameters was a better prediction system. This assessment can be applied to CRC patients for individualized prevention and treatment.

## Supplementary Material

Supplementary figures and tables.

## Figures and Tables

**Table 1:** Prediction model for survival

Gene	Name	Coefficient
*GGT6*	gamma-glutamyltransferase 6	-0.004
*ACAA2*	acetyl-CoA acyltransferase 2	-0.001
*CKMT2*	creatine kinase, mitochondrial 2	-0.004
*XDH*	xanthine dehydrogenase	-0.004
*CA2*	carbonic anhydrase 2	-0.001
*PAPSS2*	3'-phosphoadenosine 5'-phosphosulfate synthase 2	-0.002
*GPX3*	glutathione peroxidase 3	0.008

## Abbreviations

CRC: colorectal cancer
TNM: tumor-node-metastasis
TCGA: The Cancer Genome Atlas
GEO: Gene Expression Omnibus
OS: overall survival
KEGG: Kyoto Encyclopedia of Genes and Genomes
FC: fold change
FDR: false discovery rate
CAN: copy-number alteration
GSEA: gene set enrichment analysis
ROC: receiver operating characteristic
TRIPOD: Transparent Reporting of a Multivariable Prediction Model for Individual Prognosis or Diagnosis
HR: hazard ratio
LASSO: least absolute shrinkage and selection operator
CI: confidence interval
PAPS: 3'-phosphoadenosine 5'-phosphosulfate
