# Supplementary material for: Prognosis Prediction for Colorectal Cancer Patients: A Risk Score Based on The Metabolic-Related Genes
Source: Int J Med Sci. 2021 Jan 1;18(3):801–10. doi: 10.7150/ijms.49576 (PMC7797536; doi:10.7150/ijms.49576)
Supplement: Supplementary file 1 — Supplementary figures and tables. [file ijmsv18p0801s1.pdf]

Table S1. TRIPOD checklist

Section/topic	Item	Development or validation?	Checklist item	Page
**Title and abstract**				
Title	1	D;V	Identify the study as developing and/or validating a multivariable prediction model, the target population, and the outcome to be predicted	1
Abstract	2	D;V	Provide a summary of objectives, study design, setting, participants, sample size, predictors, outcome, statistical analysis, results, and conclusions	2
**Introduction**				
Background and objectives	3a	D;V	Explain the medical context (including whether diagnostic or prognostic) and rationale for developing or validating the multivariable prediction model, including references to existing models	3-4
	3b	D;V	Specify the objectives, including whether the study describes the development or validation of the model, or both	3-4
**Methods**				
Source of data	4a	D;V	Describe the study design or source of data (for example, randomised trial, cohort, or registry data), separately for the development and validation data sets, if applicable	5
	4b	D;V	Specify the key study dates, including start of accrual; end of accrual; and, if applicable, end of follow-up	5
Participants	5a	D;V	Specify key elements of the study setting (for example, primary care, secondary care, general population) including number and location of centres	5
	5b	D;V	Describe eligibility criteria for participants	5
	5c	D;V	Give details of treatments received, if relevant	5
Outcome	6a	D;V	Clearly define the outcome that is predicted by the prediction model, including how and when assessed	5-6
	6b	D;V	Report any actions to blind assessment	NA

			of the outcome to be predicted	
Predictors	7a	D;V	Clearly define all predictors used in developing the multivariable prediction model, including how and when they were measured	5-6
	7b	D;V	Report any actions to blind assessment of predictors for the outcome and other predictors	NA
Sample size	8	D;V	Explain how the study size was arrived at.	5
Missing data	9	D;V	Describe how missing data were handled (for example, complete-case analysis, single imputation, multiple imputation) with details of any imputation method	5
Statistical analysis methods	10a	D	Describe how predictors were handled in the analyses	5-7
	10b	D	Specify type of model, all model-building procedures (including any predictor selection), and method for internal validation	5-7
	10c	V	For validation, describe how the predictions were calculated	6-7
	10d	D;V	Specify all measures used to assess model performance and, if relevant, to compare multiple models	6-7
	10e	V	Describe any model updating (for example, recalibration) arising from the validation, if done	NA
Risk groups	11	D;V	Provide details on how risk groups were created, if done	6
Development *v* validation	12	V	For validation, identify any differences from the development data in setting, eligibility criteria, outcome, and predictors	5
**Results**				
Participants	13a	D;V	Describe the flow of participants through the study, including the number of participants with and without the outcome and, if applicable, a summary of the follow-up time. A diagram may be helpful	5,8
	13b	D;V	Describe the characteristics of the participants (basic demographics,	5,8

			clinical features, available predictors), including the number of participants with missing data for predictors and outcome	
	13c	V	For validation, show a comparison with the development data of the distribution of important variables (demographics, predictors and outcome).	NA
Model development	14a	D	Specify the number of participants and outcome events in each analysis	8
	14b	D	If done, report the unadjusted association between each candidate predictor and outcome	NA
Model specification	15a	D	Present the full prediction model to allow predictions for individuals (that is, all regression coefficients, and model intercept or baseline survival at a given time point)	8-10
	15b	D	Explain how to use the prediction model	8-10
Model performance	16	D;V	Report performance measures (with CIs) for the prediction model	8,9
Model updating	17	V	If done, report the results from any model updating (that is, model specification, model performance)	NA
**Discussion**				
Limitations	18	D;V	Discuss any limitations of the study (such as nonrepresentative sample, few events per predictor, missing data)	13
Interpretation	19a	V	For validation, discuss the results with reference to performance in the development data, and any other validation data	11,12
	19b	D;V	Give an overall interpretation of the results, considering objectives, limitations, results from similar studies, and other relevant evidence	11,12
Implications	20	D;V	Discuss the potential clinical use of the model and implications for future research	13
**Other information**				
Supplementary information	21	D;V	Provide information about the availability of supplementary resources, such as study protocol, Web calculator,	5,6

			and data sets	
Funding	22	D;V	Give the source of funding and the role of the funders for the present study	14

D: development; V: validation; NA: not available.
